# Electroacupuncture for the treatment of cancer pain: a systematic review and meta-analysis of randomized clinical trials

**DOI:** 10.3389/fpain.2023.1186506

**Published:** 2023-05-15

**Authors:** Junning Zhang, Weizhen Wu, Yuehan Ren, Yi Yuan, Liqun Jia

**Affiliations:** ^1^ Graduate School, Beijing University of Chinese Medicine, Beijing, China; ^2^ Department of Oncology of Integrative Chinese and Western Medicine, China-Japan Friendship Hospital, Beijing, China

**Keywords:** electroacupuncture, cancer pain, systematic review, complementary and alternative medicine, meta-analysis, rct

## Abstract

**Objective:**

This paper aims to review the current evidence on electroacupuncture as an effective and safe therapy for cancer pain management.

**Methods:**

Five databases were searched from their inception through November 11, 2022. Only the randomized controlled trials that meet the eligibility criteria were finally included in the study. Literature screening and data extraction were performed independently by two reviewers, and RevMan 5.3 used for meta-analysis.

**Results:**

A total of 17 RCTs met our inclusion criteria. We used 8 indicators to estimate the meta-analysis results, most of which proved statistically significant, including VAS scores, NRS scores, and KPS scores. To be specific, VAS scores (MD = −1.41, 95% CI: −2.42 to −0.41, *P* = 0.006) and NRS scores (MD = −1.19, 95% CI: −1.72 to −0.66, *P* < 0.0001) were significantly lower in the treatment group compared to the control group. The treatment group's KPS scores (MD = 5.48, 95% CI: 3.27 to 7.69, *P* < 0.00001) were higher than those of the control group. Also, in the treatment group, the number of burst pain (MD = −2.66, 95% CI: −3.32 to −1.99, *P* < 0.00001) and side effect rates (RR = 0.51, 95% CI: 0.39 to 0.67, *P* < 0.00001) greatly reduced, while the response rate (RR = 1.17, 95% CI: 1.09 to 1.26, *P* < 0.0001) significantly increased compared to the control group.

**Conclusion:**

This study demonstrates the advantages of electroacupuncture in the treatment of cancer pain. Meanwhile, rigorous RCTs should be designed and conducted in the future to further demonstrate the exact efficacy of electroacupuncture.

**Systematic Review Registration:**

https://www.crd.york.ac.uk/PROSPERO/, identifier CRD42022376148.

## 1. Introduction

Cancer is a large group of diseases that can start in almost any organ or tissue of the body when abnormal cells grow uncontrollably and spread to other organs (1). It is a leading cause of death worldwide, accounting for nearly 10 million deaths in 2020, or nearly one in six deaths (2). One of the major challenges in cancer treatment is pain management. Cancer pain can be caused by a tumor compressing or infiltrating nearby body parts, treatments and diagnostic procedures, skin, nerve and other changes caused by a hormone imbalance or immune response (3). Up to 2022, the overall prevalence of cancer pain was 44.5% (4). Cancer pain relief is an important aspect of cancer care, which can be achieved by using a combination of pharmacological and non-pharmacological interventions, such as opioids, adjuvant drugs, nerve blocks, acupuncture, massage and psychotherapy (5), although the most widely-used management is analgesic agents alone (6).

The World Health Organization (WHO) proposed a three-step analgesic ladder for cancer pain relief in 1986, which recommends using different types of drugs according to the intensity of pain. The first step involves using non-opioid analgesics (such as paracetamol or ibuprofen) for mild pain; the second step involves using weak opioids (such as codeine or tramadol) for moderate pain; and the third step involves using strong opioids (such as morphine or fentany l) for severe pain (7).

However, this approach has some limitations and drawbacks. First, many patients do not receive adequate pain relief due to under prescription or undertreatment of opioids (8). Second, opioids have significant side effects such as constipation, nausea, sedation, respiratory depression and addiction (9). Third, opioids are often inaccessible or unaffordable in low- and middle-income countries due to regulatory barriers and supply issues (10). Therefore, alternative therapies such as acupuncture have been increasingly used to complement or replace conventional pharmacological interventions (11).

Over the past few decades, acupuncture has gained increasing popularity in the Western world as a complementary therapy for a range of conditions, including pain management (12). One promising therapy that has been increasingly used for cancer pain is electroacupuncture (13). Electroacupuncture is a form of acupuncture that involves applying electrical currents to needles inserted at specific points on the body (14). A growing body of research has demonstrated the effectiveness of electroacupuncture in managing cancer pain. For example, a systematic review of acupuncture in cancer care found that acupuncture was effective in reducing pain in patients with cancer pain, and that electroacupuncture may have additional benefits in reducing pain intensity and duration (15). Electroacupuncture has several advantages over conventional acupuncture. For instance, it can stimulate deeper tissues and produce stronger analgesic effects by activating different types of nerve fibers (16). It can also reduce the number and duration of needles required and allow more precise control over stimulation intensity and frequency (17). Furthermore, electroacupuncture has a low cost and few side effects compared to pharmacological treatments (18).

In summary, this paper aims to review the current evidence on electroacupuncture as an effective and safe therapy for cancer pain management. We will discuss its mechanisms of action, clinical applications, and future directions. We hope that this paper will provide useful information for clinicians and researchers who are interested in electroacupuncture as an alternative or complementary option for cancer pain treatment.

## 2. Methods

PROSPERO registration has been completed in November 2022 with the registration number CRD42022376148. More details available at https://www.crd.york.ac.uk/PROSPERO/. Before the start of our study, considering that the pre-defined outcome indicators could not cover the main outcomes currently observed for cancer pain, we adjusted the implementation protocol by adding NRS (Numerical Rating Scale), times of burst pain and treatment response rate as our observed indicators. The modified protocol was reviewed and approved by two reviewers (LQJ and JNZ). Reporting standard followed PRISMA statement (see Supplementary Material).

### 2.1. Data sources

Three English-language databases and two Chinese-language databases were searched from their inception through November 11th, 2022: China National Knowledge Infrastructure (CNKI), Wanfang Database for Chinese Technical Periodicals, PubMed, Web of Science, and Cochrane Central Registry of Controlled Trials (CENTRAL). We use the MeSH term, title, and abstract to search the three English databases were: (electroacupuncture or electro-acupuncture) AND (“cancer” OR “tumour” OR “neoplasm”) AND (“pain OR ache OR cancer pain”) AND (“randomized controlled trial”). The keywords were then translated into Chinese and searched in the two remaining Chinese databases. After reading the full text, we collected them together in the ZOTERO database, in which the repetitive literature was removed.

### 2.2. Study selection

RCTs were included if electroacupuncture was used as the only intervention or as an adjunct to another standard treatment for cancer pain and the control group received the same concomitant treatment as the electroacupuncture group. We ignored whether the included studies used the correct randomization method, allocation concealment, and blinding. There were no language restrictions. Trials that used comparative treatments/groups that were expected to have similar effects to electroacupuncture (moxibustion, transcutaneous electrical acupoint stimulation, acupoint injection, laser irradiation, cupping, Tuina, etc.) or that used Chinese herbal medicine were excluded. Trials that studied cancer pain mixed with other types of pain and trials that were performed on patients during or a few days after surgery for malignancy were also excluded. Trials were also excluded if their results were not related to cancer pain.

The studies we included involved at least one of the following outcomes.

Primary Outcome Indicators.

• VAS (Visual Analogue Scale);
• NRS (Numerical Rating Scale);
• NPS (Neuropathic Pain Scale);
• BPI (Brief Pain Inventory);
• KPS (Karnofsky Performance Status).

Secondary Outcome Indicators.

• Times of burst pain;
• Treatment response rate;
• Side effect rates.

In addition, considering the differences in the definitions of treatment response rate among studies, we defined the efficiency rate. The treatment response rate was determined by the degree of pain relief, and the efficacy index = (pre-treatment NRS score—post-treatment NRS score)/pre-treatment NRS score × 100%. Complete remission (CR): efficacy index was 91%–100%; apparent remission (AR): efficacy index was 61%–90%; partial remission (PR): efficacy index was 31%–60%. No remission (NR): efficacy index <31%. Effective cases were CR + AR + PR. We included the results of related studies for analysis if their outcome measures approximated our definition.

### 2.3. Methodology quality assessment and data extraction

The risk of bias was assessed using the following criteria from the Cochrane classification: random sequence generation, allocation concealment, blinding of participants and staff, blinding of outcome assessment, incomplete outcome data, selective reporting and other types of bias. The authors classified studies as “low risk” (L), “unclear risk (U)” and “high risk” (H) (19).

All articles were read by two independent reviewers (JNZ and WZW) who independently assessed the study selection, methodology quality assessment, and data extraction process, then we cross-checked the data. Any disagreements were resolved by discussion or consultation with a third independent reviewer (YHR).

### 2.4. Data analysis

If the study contained insufficient information, we tried to communicate with the lead author to obtain accurate data. RevMan 5.3 software provided by the Cochrane Collaboration Network was used for the meta-analysis. In this study we choose random effects model for our analysis. The risk ratio (RR) was used for the dichotomous variables, the mean difference (MD) was used for the continuous variables, and the 95% confidence interval (CI) was used for each effect quantity.

The *Chi^2^* test was used for heterogeneity among the results of the included studies. In this study we used the following *I*² thresholds:

• *I²*:0%–40%: probably insignificant;
• *I²*:30%–60%: may represent moderate heterogeneity;
• *I²*:50%–90%: may represent significant heterogeneity;
• *I²*:75%–100%: considerable heterogeneity.

The statistical value of *I²* depends on the size of its influence, and the strength for the evidence of heterogeneity (e.g., the *p*-value of the *Chi^2^* test). We performed sensitivity analysis on the comparison results with very high heterogeneity. The impact of clinical and statistical heterogeneity on the results will be considered when discussing the results of the analysis.

## 3. Results

### 3.1. Search results and study description

**Figure 1:**
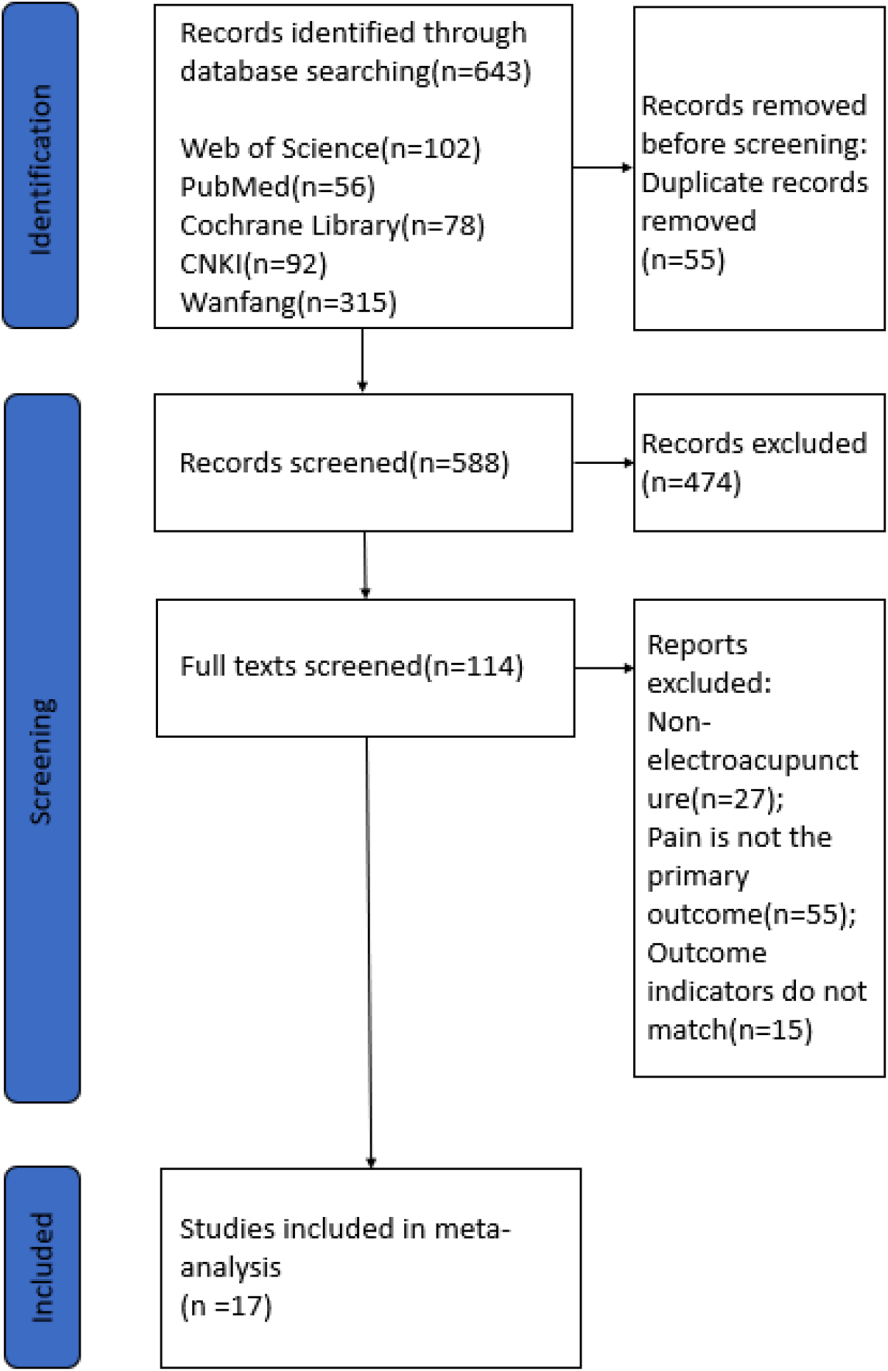
Flowchart of the literature review and selection process.

The literature search initially identified 643 articles. Of these, 55 duplicate articles and 571 articles that were not relevant to the selection criteria were excluded. Finally, 17 studies were included in our meta-analysis (Figure 1), and the characteristics of all included RCTs are shown in Table 1 (20%–36%). A total of 1,275 cases of cancer pain were included, with study sample sizes ranging from 7 to 360. 11 trials were published in Chinese and another 6 in English. 4 trials used analgesics (three-step analgesic ladder) as comparators (26, 28, 31, 34), 3 trials applied sham controls including minimal or superficial needling at non-acupuncture points (21, 22, 25), and the remaining few trials used other conventional Western medications or usual care.

**Table 1:** Basic information of the included literature.

First author (year)	Type of cancer	Sample sizes		Interventions		Acupuncture points selection	Session frequency and duration	Main outcomes	Electroacupuncture method. Did the research report "deqi"?	Adverse events
		T	C	T	C					
Saraswati 2020	Cervical cancer	14	14	EA + C	Paracetamol and codeine	ST36,SP6,LI4,LR3	30 min qd 3 weeks	1 VAS 2 QOL	Disperse dense wave, frequency of 2/20–25 Hz. n.r.	n.r.
Greenlee 2016	Breast cancer	25	23	EA	Sham EA	GB34,ST36,LI4,LI10	30 min qw 12 weeks	1 BPI 2 NPS	2 Hz of mixed pulsatile intervals. deqi	Swelling, and bruising
Minton 2007	Not clear	3	4	EA	Placebo (sham) needling	n.r.	30 min qw 12 weeks	NPS	Alternating current at 80 and 2 Hz. n.r.	n.r.
Mao 2020	Not clear	145/143	72	EA/AA	Usual care	n.r.	30 min Patients received 10 treatments over 10 weeks	BPI	2 Hz. deqi	Swelling, and bruising
Wong 2006	Non-small cell lung cancer	13	12	EA	Sham EA	LI4,GB34, GB36,TE8	30 min bid 7 days	VAS	Alternating current at 60 Hz. deqi	No
Rostock 2013	Various	14	17	EA	Placebo	LV3,SP9,GB41,GB34,LI4,LI11,SI3,HT3.	15 min Patients received 9 treatments over 3 weeks	1 NRS 2 EORTCQLQ-C30	Rectangular currents and high amplitude waves, frequency of 50 Hz. deqi	n.r.
Peng Jie 2012	Not clear	23	24	EA + C	Drug (three-step analgesic ladder)	LI4,PC6,ST36,SP6	30 min qd 7 days	Response rate	Disperse dense wave, frequency of 4/20 Hz. deqi	n.r.
Xu Chuting 2020	Lung cancer	35	35	EA + C	Oxycodone	SiGuan,ST36,HT7,SJ5,back-shu points	30 min qd 4 weeks	1 NPS 2 Response rate	Disperse dense wave, frequency of 15/100 Hz, current 8 ± 2 mA. n.r.	1 Insomnia 2 Constipation 3 Nausea and vomiting 4 Dizzy 5 Rash, and itching
Wang Ying 2017	Non-small cell lung cancer	30	30	EA + C	Oxycodone sustained-release tablets	LI4,PC6,ST36,SP6	30 min qd 2 weeks	1 NRS 2 EORTCQLQ-C30 3 Response rate4 4 Burst pain	Continuous wave, frequency of 15 Hz. deqi	1 Constipation 2 Nausea and vomiting 3 Dizzy 4 Rash, and itching
Yang Zhiling 2021	Various	30	30	EA + C Drug	Drug (three-step analgesic ladder)	LU6,LR3,SiGuan,ST36,PC6	20–30 min qd 7 days	NRS	Disperse dense wave, frequency of 2/100 Hz. deqi	1 Constipation 2 Nausea and vomiting
Wang Hui 2018	Lung cancer	30	30	EA + C	Oxycodone sustained-release tablets	Based on syndrome differentiation and disease differentiation	30 min qd 2 weeks	1 Response rate 2 Burst pain 3 EORTCQLQ-C30	Continuous wave, frequency of 15 Hz. deqi	1 Constipation 2 Nausea and vomiting 3 Rash, and itching
Zhang Yixiao 2020	Pancreatic cancer	19	18	EA + C	Celiac plexus ablation	Based on syndrome differentiation and disease differentiation	30 min qod 3 weeks	1 VAS 2 QOL	Disperse dense wave, frequency of 2/100 Hz, current 0.5–4 mA. n.r.	No
Wu Hui 2021	Pancreatic cancer	50	50	EA + C	Celiac plexus ablation	PC6,ST36,BL20,LI4,BL23,SP6	30 min qod 3 weeks	VAS	Disperse dense wave, frequency of 2/100 Hz. n.r.	n.r.
Zhu Weijian 2021	Colon cancer	43	43	EA + C	Accelerated rehabilitation surgery program	ST36(both),ST37(both)	30 min qd 5 days	VAS	Continuous wave, frequency of 2 Hz, current 1–2mA. deqi	Yes (but not specifically reported)
Wang Can 2019	Various	40	40	EA + C	Hydromorphone	LI4,LR3,PC6,RN6,LI11,ST36,SP6, AShi point	30 min qd 2 weeks	1 NRS 2 KPS 3 Response rate 4 Burst pain	15 Hz. deqi	1 Constipation 2 Nausea and vomiting 3 Dizzy 4 Rash, and itching 5 Dysuria, and urinary retention
Shen Lufei 2016	Lung cancer	50	50	EA + C	Drug (three step analgesic ladder)	Based on syndrome differentiation and disease differentiation	30 min qd 4 weeks	1 NRS 2 Response rate	Main acupoints: dense wave, frequency of 10 Hz. Auxiliary acupoints: dense wave, frequency of 100 Hz. n.r.	n.r.
Wang Yanchun 2014	Not clear	38	38	EA + C	Drug (three-step analgesic ladder)	Based on syndrome differentiation and disease differentiation	15 min qod 30 days	1 NRS 2 KPS 3 Response rate	Continuous wave, frequency of 2 Hz, current 1 mA. deqi	1 Constipation 2 Nausea and vomiting 3 Dizzy 4 Dysuria, and urinary retention

T, treatment group; C, control group; EA, electroacupuncture; AA, auricular-acupuncture; n.r., not reported; VAS, visual analogue scale; NRS, numerical rating scale; NPS, neuropathic pain scale; BPI, brief pain inventory; KPS, karnofsky performance status; EORTC QLQ-C30, European organization for research and treatment of cancer quality of life questionnaire; QoL, quality of life; qd, once a day; qw, once a week; bid, twice a day; qod, once every other day.

Zusanli (ST36), Hegu (LI4), Sanyinjiao (SP6), and the extraordinary point Siguan were the most frequently used acupoints. Eleven studies reported “deqi” (21, 23–26, 29–32, 34, 36), a sensation of needling perceived as soreness, numbness, or distension, which is usually achieved by manipulating acupuncture needles to obtain the desired therapeutic effect, and seven other studies did not mention this effect (20, 22, 27, 28, 33, 35). In most studies, patients were treated for 30 min per session. The duration of time patients received electroacupuncture treatment ranged from 1 to 12 weeks. In terms of electroacupuncture waveforms, six studies used disperse dense wave (20, 26, 27, 33–35), four studies used continuous waves (30–32, 36), and one study used only dense waves (28), with the remaining trials not specified; in terms of electroacupuncture frequency, it ranged from 2 hz to 100 hz, in terms of applying electroacupuncture current intensity, only a few studies specifically reported the intensity of the current used (27, 31, 35, 36), most of the other trials stated the maximum intensity within the patients’ tolerance.

### 3.2. Risk of bias

**Figure 2:**
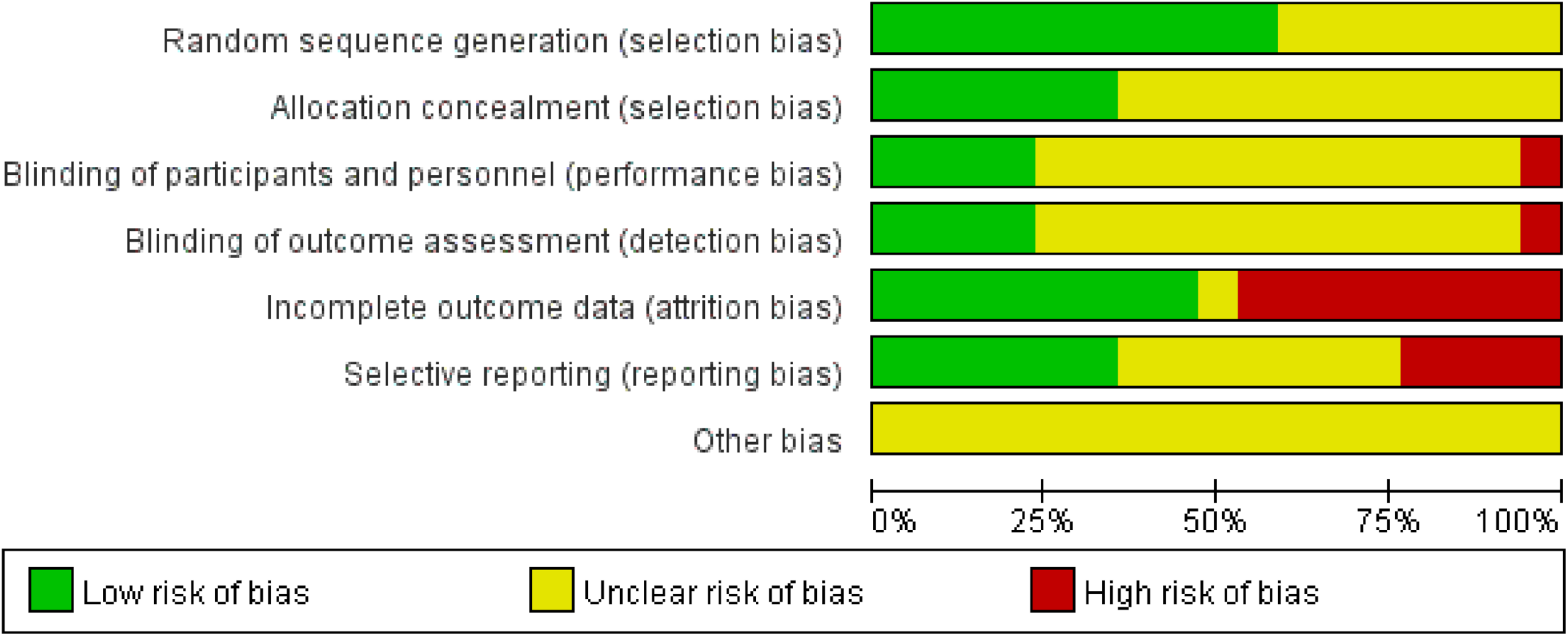
Assessment of cochrane risk of bias presented as percentages across all included studies.

**Figure 3:**
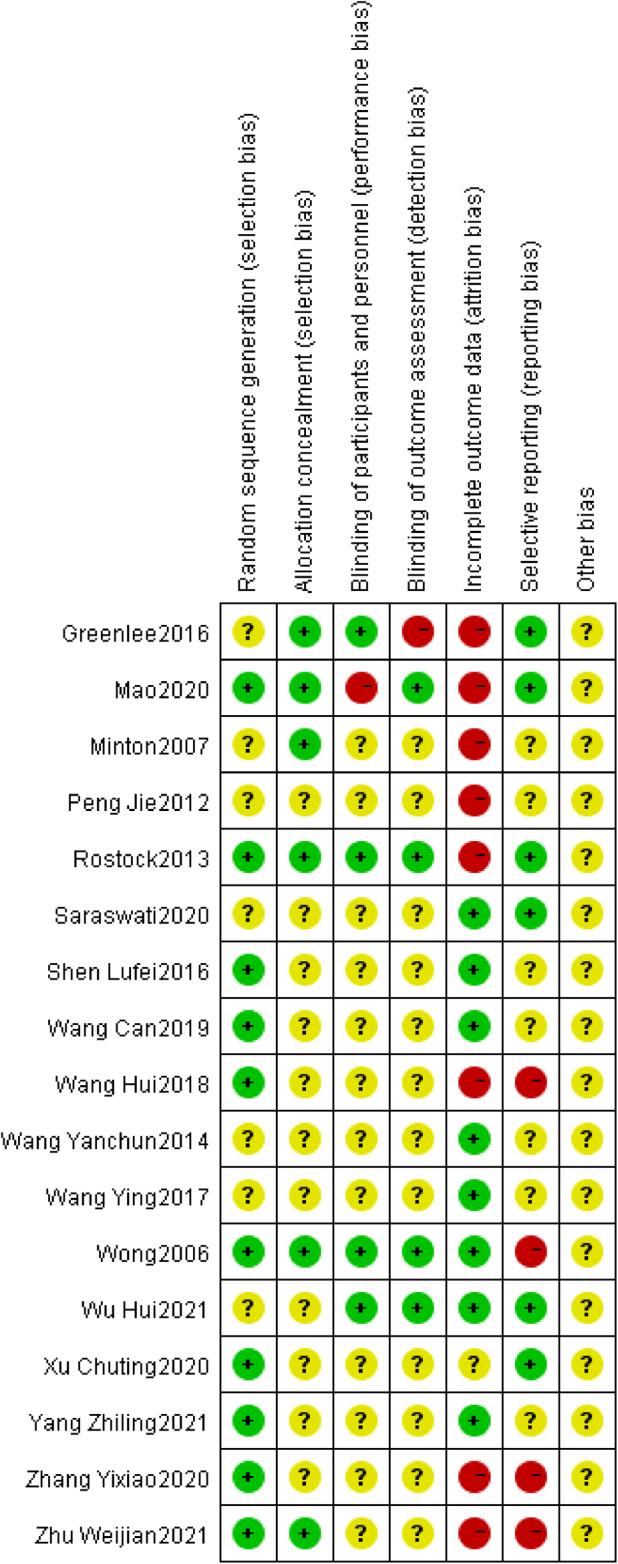
Cochrane risk of bias summary for each included study.

Risk of bias graph could be founded in Figure 2. Risk of bias summary could be located in Figure 3.

#### 3.2.1. Selection bias

Of the 17 RCTs included, 7 RCTs did not describe problems associated with the randomization process (20–22, 26, 31–33). 7 RCTs used a table of random numbers (27–30, 34–36), Mao et al. performed randomization by a secure system with full allocation hiding (23), and Wong et al. used a computer-generated random sequence (25). Rostock et al. performed randomization by nonstratified block with randomly varying lengths (24). 6 studies used allocation hiding and reported the details (21–25, 36). The remaining trials did not report specific methods of allocation concealment implementation.

#### 3.2.2. Performance bias

Because the RCTs included in this study involved treatment and control groups that differed significantly in the form and manipulation of the intervention, it was more difficult to apply blinding to participants or personnel, and only four RCTs used correct blinding for participants and personnel (21, 24, 25, 33).

#### 3.2.3. Detection bias

Only 4 of the 17 RCTs included implemented correct blinding of assessors for outcome indicators (23–25, 33), 1 study did not use blinding of outcome assessment (21), and the remaining trials did not report the specific method of blinding implementation.

#### 3.2.4. Attrition bias

Eight RCTs reported exit or withdrawal information appropriately (21–24, 26, 30, 35, 36), however, missing data were not addressed, so they were rated as high risk of bias in the attrition bias evaluation. Eight trials did not have missing data (20, 25, 28, 29, 31–34). One trial did not report the presence or absence of missing data and was judged to be at uncertain risk (27).

#### 3.2.5. Reporting bias

Of the 17 RCTs included, 6 RCTs reported that their study teams pre-defined the study protocol and were therefore evaluated as low risk in reporting bias (20, 21, 23, 24, 27, 33). 4 RCTs did not report the full study protocol and were therefore judged to be at high risk (25, 30, 35, 36). The remaining 7 trials did not report relevant information and were therefore evaluated as unclear in the risk of bias judgment.

#### 3.2.6. Other bias

This study evaluated publication bias, study design, and confounding bias in the included randomized controlled trials, and the risk of other bias was not clear for all trials because the content of the relevant information was not sufficient.

### 3.3. Primary outcome indicators

#### 3.3.1. VAS score

**Figure 4:**
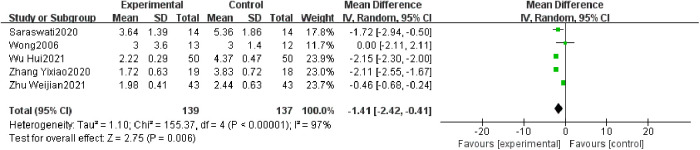
Forest plot of VAS score.

Five studies reported post-treatment VAS scores (20, 25, 33, 35, 36), with 139 cases in the treatment group and 137 cases in the control group overall. Heterogeneity tests for meta-analysis showed a statistically significant difference between studies with *I*² = 97%. VAS scores were lower in the treatment group than in the control group, with a statistically significant difference (276 participants, MD = −1.41, 95% CI: −2.42 to −0.41, *P* = 0.006; see Figure 4).

#### 3.3.2. NRS score

**Figure 5:**
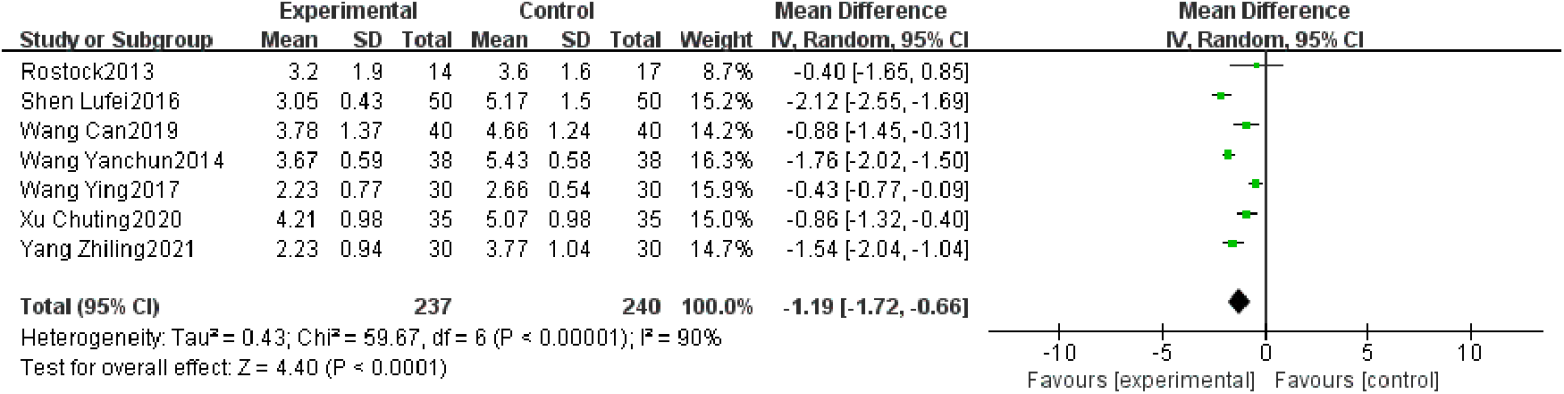
Forest plot of NRS score.

Seven studies reported post-treatment NRS scores (24, 27–29, 31, 32, 34), with 237 cases in the treatment group and 240 cases in the control group overall. Heterogeneity tests for meta-analysis showed that *I*² = 90%. NRS scores were lower in the treatment group than in the control group, with a statistically significant difference (477 participants, MD = −1.19, 95% CI: −1.72 to −0.66, *P* < 0.0001; see Figure 5).

#### 3.3.3. Other pain scores

**Figure 6:**
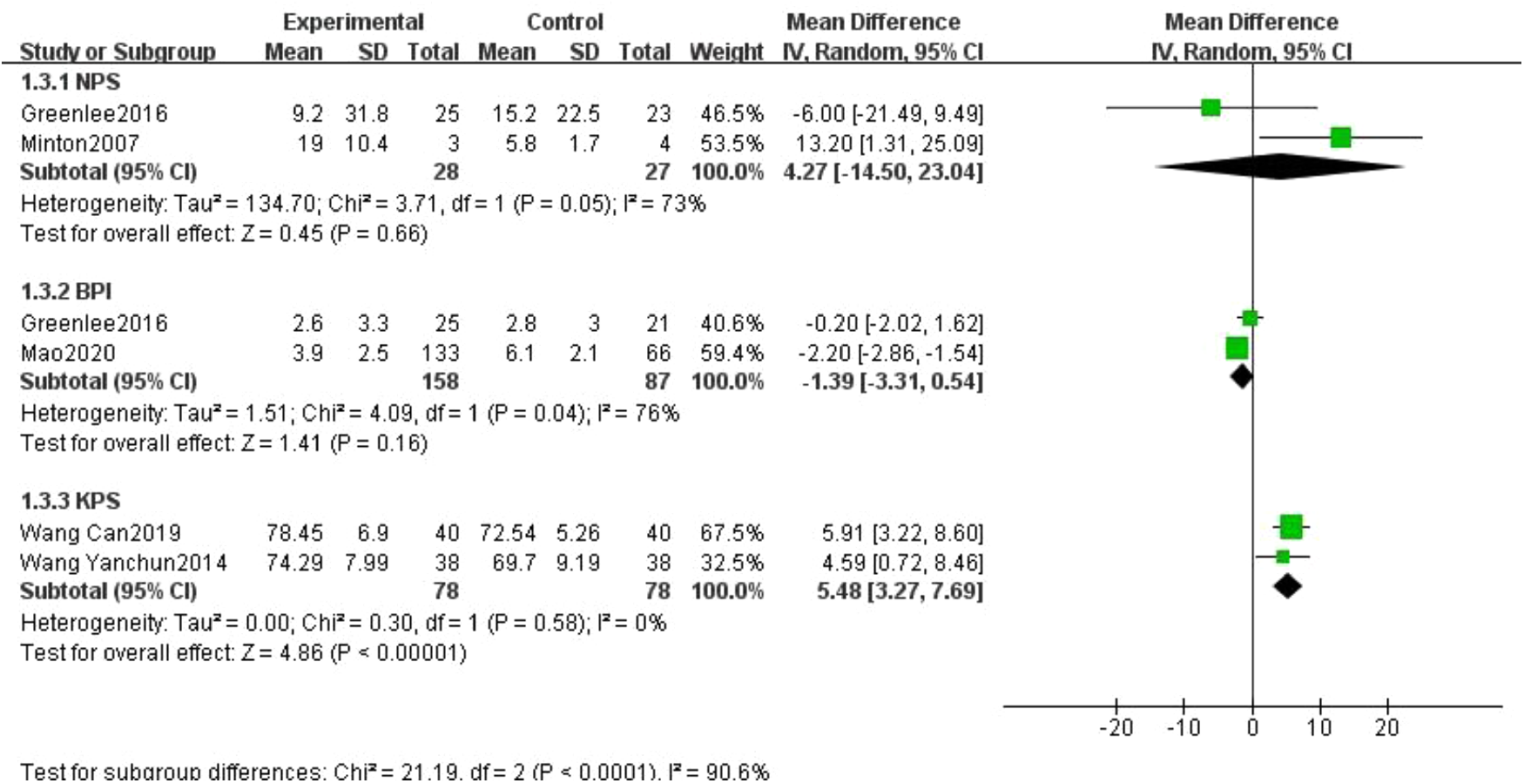
Forest plot of other pain score.

The Neuropathic Pain Scale (NPS) (21, 22), Brief Pain Inventory (BPI) (21, 23) and Karnofsky performance status (KPS) (29, 31) were reported in two studies each, so they were combined in a single icon and analyzed using a random-effects model in this study. Heterogeneity tests for meta-analysis of the NPS scores showed that *I*²* *= 73%, the NPS scores in the treatment group were not lower than those in the control group, the difference was not statistically significant and did not indicate that the treatment group improved the NPS scores better than the control group (55 participants, MD = 4.27, 95% CI: −14.50 to 23.04, *P* = 0.66;). Heterogeneity tests for meta-analysis of BPI scores showed that *I*² = 76%, and the BPI scores were statistically lower in the treatment group than in the control group (245 participants, MD = −1.39, 95% CI: −3.31 to 0.54, *P* = 0.16). KPS scores were higher in the treatment group than in the control group, indicating a better improvement in KPS scores in the treatment group than in the control group (156 participants, MD = 5.48, 95% CI: 3.27 to 7.69, *P* < 0.00001; see Figure 6).

### 3.4. Secondary outcome indicators

#### 3.4.1. Burst pain

**Figure 7:**
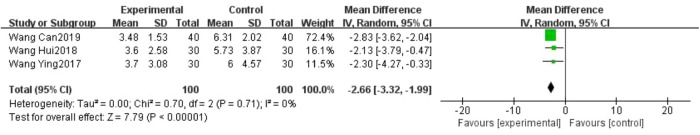
Forest plot of times of burst pain.

Three studies reported the times of burst pain (29, 30, 32). The combined statistical results showed that the incidence of burst pain was lower in the treatment group than in the control group, with a statistically significant difference (200 participants, MD = −2.66, 95% CI: −3.32 to −1.99, *P* < 0.00001; see Figure 7).

#### 3.4.2. Response rates

**Figure 8:**
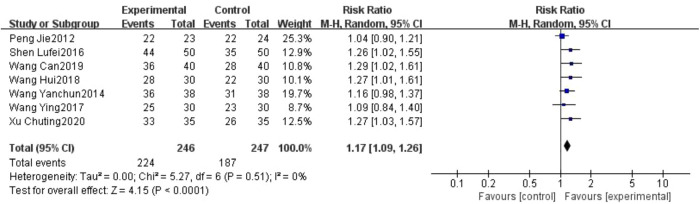
Forest plot of the response rates.

Seven studies reported response rates for pain relief after treatment (26–32). Of 246 cases in the treatment group, 224 had a valid response; of 247 cases in the control group, 187 had a valid response. The overall response rate of the treatment group was better than the control group, with a statistically significant difference, (493 participants, RR = 1.17, 95% CI: 1.09 to 1.26, *P* < 0.0001; see Figure 8).

#### 3.4.3. Side effect rates

**Figure 9:**
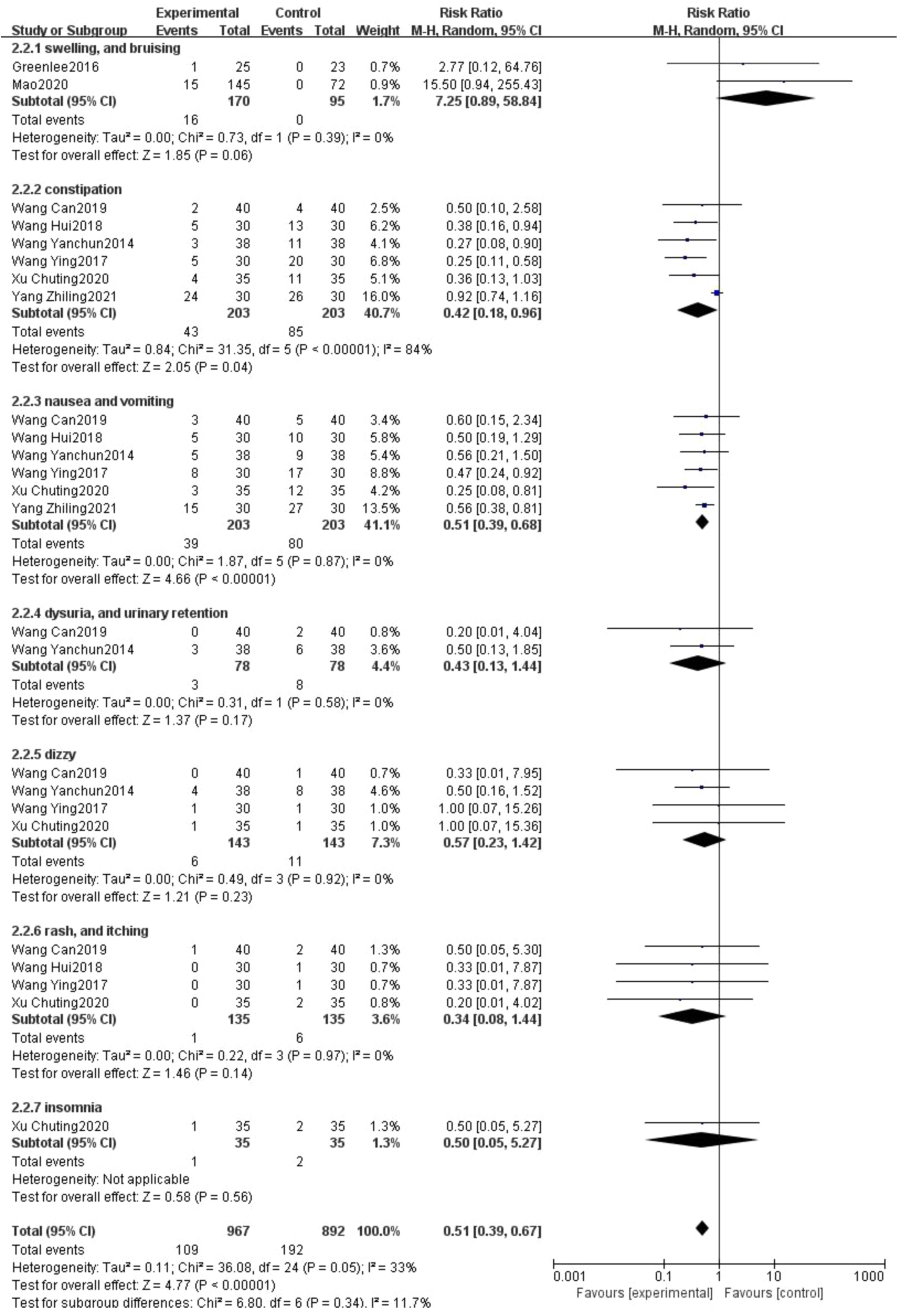
Forest plot of side effect rates.

Side effects mainly included swelling, bruising, constipation, nausea, vomiting, dysuria, urinary retention, dizziness, rash, itching and insomnia. 2 studies reported the number of cases of swelling and bruising (21, 23), 6 studies reported the number of cases of constipation (27, 29–32, 34), 6 studies reported the number of cases of nausea and vomiting (27, 29–32, 34), 2 studies reported the number of cases of difficulty urinating and urinary retention (29, 31), 4 studies reported the number of cases of dizzy (27, 29, 31, 32), 4 studies reported the number of cases of rash and itching (27, 29, 30, 32) and 1 study reported the number of cases of insomnia (27). The combined statistical results showed that the incidence of constipation (406 participants, RR = 0.42, 95% CI: 0.18 to 0.96, *P* = 0.04) and nausea and vomiting (406 participants, RR = 0.51, 95% CI: 0.39 to 0.68, *P* < 0.00001) were significantly lower in the treatment group compared with the control group, but the incidence of swelling, and bruising (265 participants, RR = 7.25, 95% CI: 0.89 to 58.84, *P *= 0.06) was higher; several other adverse reactions were not statistically significant, perhaps due to the small sample size. But overall, adverse reactions occurred in 109 out of 967 cases in the treatment group and in 192 out of 892 cases in the control group. The number of adverse reactions that occurred in the treatment group was lower than in the control group, and the difference was statistically significant, (1,859 participants, RR = 0.51, 95% CI: 0.39 to 0.67, *P* < 0.00001; see Figure 9).

### 3.5. Sensitivity analysis

The results of the quantitative synthesis of our involved outcome indicators showed high heterogeneity for only two outcome indicators, VAS score and NRS score, for which we performed sensitivity analysis. While the quantitative synthesis results of the other outcome indicators had low heterogeneity or involved only 2 trials, so sensitivity analysis could not be performed.

Sensitivity analyses showed that we excluded each of the five trials involved in the VAS score and found a significant decrease in inter-study heterogeneity after Zhu Weijian2021 (36) was excluded, and the combined results of the four trials showed *I*² = 32%, MD = −2.07, 95% CI: −2.38 to −1.76, *P* < 0.00001.

The results of our sensitivity analysis of the NRS scores showed that combining four of the trials (24, 27, 29, 32) showed *I*² = 6%, MD = −0.63, 95% CI: −0.89 to −0.38, *P* < 0.00001 and combining the other three (28, 31, 34) showed *I*² = 38%, MD = −1.81, 95% CI: −2.09 to −1.53, *P* < 0.00001. Heterogeneity was also greatly reduced after splitting the analysis into these two parts.

## 4 . Discussion

The purpose of this systematic review was to critically assess the effectiveness of electroacupuncture for cancer pain by updating and refining new evidence. The current meta-analysis based on 17 studies showed that electroacupuncture was effective in relieving cancer pain in cancer patients, and that compared with controls, electroacupuncture for cancer pain resulted in lower several major pain scores such as VAS scores and NRS scores, lower rates of adverse effects such as constipation, nausea, and vomiting, and fewer times of burst pain.

The mechanism of electroacupuncture for cancer pain is not fully understood, but it may involve multiple pathways that modulate pain signaling and inflammatory responses at different levels of the nervous, immune, and endocrine systems (16). Han et al. revealed the neural mechanism of broad-spectrum analgesia by electroacupuncture: electroacupuncture stimulates specific acupuncture points on the body with electrical impulses, activating surrounding nerve fibers that transmit signals to the spinal cord and brain, which in turn activates the endogenous opioid system, releasing natural analgesics such as endorphins, enkephalins, and dynorphins in the brain and spinal cord (37). In addition to activating the neurological secretion of opioid substances, electroacupuncture acts synergistically with endogenous opioids to inhibit astrocyte activation by suppressing spinal glial fibrillary acidic protein (GFAP) expression, thereby reducing bone cancer pain in rats (38). It's also reported to relieve morphine tolerance of rats with breast cancer by promoting the internalization of µ-opioid receptor (MOR) and Rab5, a protein involved in endocytosis, locating in the locus coeruleus region (39). As for immunology pathways, in a study of a rat model of prostate cancer bone metastases, electroacupuncture was found to inhibit pro-inflammatory cytokines, such as IL-1β, which attenuate nociceptive receptor sensitivity and inhibit pain transmission (40). Electroacupuncture can also directly downregulate the expression of nociceptive receptors in a rat model of cancer pain, such as P2X3 receptors in the dorsal root ganglion of rats (41). Electroacupuncture can modulate immune cells, such as macrophages (42), mast cells (43) and T cells (44), to reduce pain-causing substances produced by inflammation and tissue injury. These mechanisms can work in concert to reduce the intensity of cancer pain and improve the quality of life of cancer survivors.

We found some aspects that need improving in the clinical research of our topic. Most of the included trials lacked a description of the electroacupuncture treatment protocol, such as the sensation of “deqi”, the depth of needling, and the corresponding parameters of electroacupuncture; in addition, the optimal dose of electroacupuncture is not known. This not only limits the quality of the studies, but also raises the question of whether electroacupuncture adequately elicited a functional response and exerted sufficient therapeutic effects to control cancer pain in the included trials. Therefore, we strongly recommend that researchers should clearly validate and report the sensation of gaining breath and the depth of needling in future studies and unify electroacupuncture parameters as much as possible. In addition, future RCTs should focus on exploring the effective dose of electroacupuncture for cancer pain. To achieve this goal, future studies should include sufficiently large samples, extend the duration of treatment and follow-up, and standardize the efficacy evaluation system. Several RCTs had no adverse effects (25, 35), which seems to conflict with research ethics and guidelines for reporting clinical trials. Acupuncture is not completely free of adverse reactions (45). Failure to report adverse effects in clinical trials would create inaccuracies regarding the safety of this treatment. Subjective symptom alleviation of patients' initial cancer pain status was reported in all included RCTs. Due to the absence of objective assessment methods, studies on pain frequently rely on patient testimonies. A methodical, scientific approach is consequently required for the evaluation of cancer pain. Such a strategy must incorporate a thorough study of the available literature, expert judgment and consensus, a strict translation procedure, and thorough validation (46). The application of such a strategy could raise the bar for evaluating cancer pain.

However, this meta-analysis has several limitations. First, the treatment protocols and selected parameters for electroacupuncture were not consistent among the included studies. Second, some of the adverse effects and the differences in NPS scores between groups were not statistically significant due to the small sample size involved, which may be related to the small number of relevant studies available. Third, methodological limitations, although we made considerable efforts to retrieve all RCTs on this issue, there were selection, performance, and detection biases in the included trials that affected the strength of the evidence and limited the internal validity of this review. Therefore, we searched 5 databases to minimize bias in this regard. Nonetheless, this review has several strengths. We are the first meta-analysis on electroacupuncture for cancer pain and include only RCTs.

In conclusion, this study demonstrates the particular advantages of electroacupuncture in the treatment of cancer pain. Rigorous RCTs should be designed and conducted in the future, and these studies need to incorporate accepted trial design and reporting standards. Specifically, they should be based on appropriate sample size calculations, use validated outcome measures, control for nonspecific effects, and adhere to modern human research ethics to further demonstrate the exact efficacy of electroacupuncture.

## Data Availability

The original contributions presented in the study are included in the article/**Supplementary Material**, further inquiries can be directed to the corresponding author/s.
